# Strain sensor on a chip for quantifying the magnitudes of tensile stress on cells

**DOI:** 10.1038/s41378-024-00719-z

**Published:** 2024-06-25

**Authors:** Yuyin Zhang, Yue Wang, Hongze Yin, Jiahao Wang, Na Liu, Songyi Zhong, Long Li, Quan Zhang, Tao Yue

**Affiliations:** 1 School of Mechatronics Engineering and Automation, Shanghai University, Shanghai, China; 2 School of Future Technology, Shanghai University, Shanghai, China; 3 Key Laboratory of Advanced Manufacturing Technology of Zhejiang Province, School of Mechanical Engineering, Zhejiang University, Hangzhou, China; 4 Shanghai Key Laboratory of Intelligent Manufacturing and Robotics, Shanghai University, Shanghai, China; 5 Shanghai Institute of Intelligent Science and Technology, Tongji University, Shanghai, China

**Keywords:** Electrical and electronic engineering, Sensors

## Abstract

During cardiac development, mechanotransduction from the in vivo microenvironment modulates cardiomyocyte growth in terms of the number, area, and arrangement heterogeneity. However, the response of cells to different degrees of mechanical stimuli is unclear. Organ-on-a-chip, as a platform for investigating mechanical stress stimuli in cellular mimicry of the in vivo microenvironment, is limited by the lack of ability to accurately quantify externally induced stimuli. However, previous technology lacks the integration of external stimuli and feedback sensors in microfluidic platforms to obtain and apply precise amounts of external stimuli. Here, we designed a cell stretching platform with an in-situ sensor. The in-situ liquid metal sensors can accurately measure the mechanical stimulation caused by the deformation of the vacuum cavity exerted on cells. The platform was applied to human cardiomyocytes (AC16) under cyclic strain (5%, 10%, 15%, 20 and 25%), and we found that cyclic strain promoted cell growth induced the arrangement of cells on the membrane to gradually unify, and stabilized the cells at 15% amplitude, which was even more effective after 3 days of culture. The platform’s precise control and measurement of mechanical forces can be used to establish more accurate in vitro microenvironmental models for disease modeling and therapeutic research.

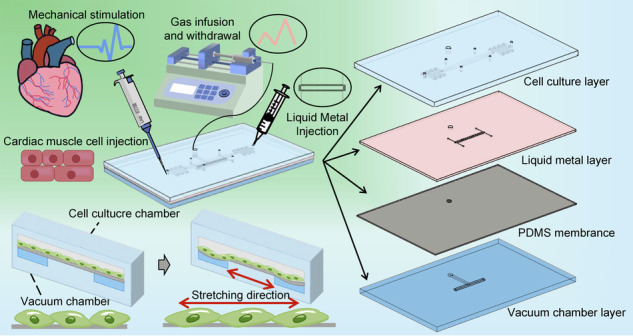

## Introduction

Multidimensional mechanical stresses are produced in human organs by the in vivo microenvironment^1–3^. The human cardiomyocyte cell line AC16 serves as a resource for mimicking heart dynamics^4^, and cardiomyocytes can be subjected to continuous mechanical stimulation to mimic in vivo dynamics. By adding external stimuli to cells cultivated in vitro, scientists have created several platforms and techniques to examine changes in cell dynamics at the cellular level. These techniques include electrical stimulation^5–8^, direct stretching^9–11^, multiple spindle stretching^12–14^, and a range of additional approaches. Clerc et al.^15^ demonstrated that transient mechanical stimulation causes cardiomyocytes to develop hypertrophic lesions. In contrast, Saucerman et al.^16^ discovered that the lack of mechanical loading of the stimulus in vivo hampered the growth of cardiomyocytes, which in turn reduced cardiac contractility. This highlights the challenge in research on using mechanical methods to prompt cells to develop their biological characteristics: accurately measuring the mechanical force applied to cells within an in vitro culture system designed for stimulation.

Microfluidic systems have been utilized in cell culture more frequently in recent years^17–20^, particularly in dynamic cell culture systems that imitate the intricate movements of bodily organs^21–24^. Microfluidic devices allow human manipulation to mimic intricate movements and possess precise dimensions that simplify the process of structural design. The elastic film deforms when the pressure in the vacuum chamber is changed by an external air valve, which is a simple way to provide tension stimulation inside the chip. However, the necessity for exact quantification versus externally induced stimuli cannot be satisfied by microfluidic chips using vacuum chambers^25,26^ and cell drums^27^. To address this need, microfluidic chips with in-situ sensors need to be designed. Sensors^28,29^ are incorporated into chips, and accurate strain can be obtained by piezoresistive^30^, piezoelectric^31–33^, and crack sensing^34^ methods. However, there are few examples of in-situ sensors on chips that include mechanical stimulation. These techniques are either not suitable for continuous monitoring or require complicated observation equipment.

In our work, we introduced a cell stretching platform that features an in-situ sensor. This platform continuously applies mechanical stimulation to cells attached to a flexible film. The stimulation is achieved by stretching and contracting the film, which is driven by the expansion and compression of a vacuum cavity inside a microfluidic chip. The in-situ sensor, which was made with liquid metal (Galinstan) and soft lithography, enables real-time monitoring of the amount of mechanical stress (5%, 10%, 15%, 20%, 25%) placed on the film (cells). The thin film sensors we designed are highly sensitive, have a linear response, and can automatically adjust to temperature changes. These benefits are achieved by harnessing the responsive nature of liquid metals and incorporating a design that uses embedded Wheatstone bridge circuits, which are known for their precise measurement capabilities. Cardiomyocytes stimulated for 24 h on the aforementioned platform showed greater activity and induced the arrangement in cell organization. Our results indicated that cardiomyocyte maturation is enhanced by mechanical stimulation in a strain magnitude-dependent manner. As we increased the amount of cyclic strain, we observed an increase in both the number and cell area of cardiomyocytes, with the most favorable results occurring at 15% cyclic strain. After more than 2 days (60 h) of incubation, cell viability significantly increased under 15% cyclic strain, and the heterogeneity of cell direction growth was largely consistent under stretching conditions. Cellular activity corresponded to a faster rate of cell proliferation and an increase in the area of individual cells; the alignment pattern corresponded to a tendency of skeletal proteins to align along the direction of stimulation. The experimental results demonstrated that our embedded in situ sensor-on-chip is a useful platform for measuring the mechanical stimulation of cells.

## Results and discussion

### Uniaxial stretching and deformation conversion

**Fig. 1:**
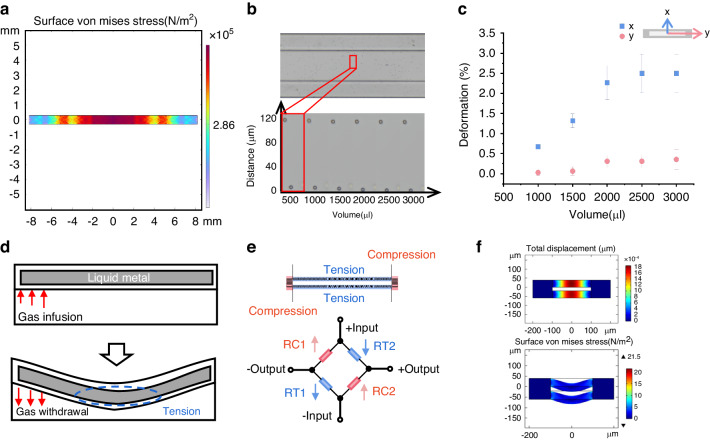
Film tensile quantification. **a** Strain map of the deformation amplitude in the film using COMSOL (Burlington, USA) and **b** particles in the film mixed with small particles at different applied pressures. **c** A typical curve illustrating the relationship between the extraction flow and membrane (horizontal and longitudinal axis directions) deformation. **d** Schematic diagram of the cross-sectional deformation of the liquid metal during the cavity deformation process. **e** Schematic layout of the in situ sensor and schematic diagram of the equivalent circuit form an equivalent Wheatstone bridge circuit. **f** Simulation of the cross-sectional deformation of the liquid metal channel

The paper studied a rectangular area (400 μm × 8000 μm) located in the middle of the air chamber in the culture chamber (shown in Fig. S2). This paper examined the direction of the polydimethylsiloxane (PDMS) membrane stretching to define the specific stress of cells during mechanical stimulation. PS beads were encapsulated in a PDMS membrane (5 μm in diameter; fluorescence: red) for a more precise quantitative study of deformation. The sequential image of particles at distinct stretching levels is shown in Fig. 1b from the initial condition to the maximum range with a constant volume decrease of 500 μL. A displacement study of the stretched membrane, specifically for contrast in various directions (in the *x*-axis direction and the *y*-axis direction), was processed using ImageJ. The reproducibility and consistency of the fabrication process were confirmed by using six sets of data from three separate chips. At a maximum withdrawal of 3000 μL, there is an almost linear deformation tendency in the *x*-axis, with a magnitude of 2.5% in the PDMS membrane. However the deformation trend in the *y*-axis tends to be steady. It is also proven that there is a general trend toward a linear rise in displacement from the membrane’s center to its edge. As shown in Fig. 1a, the stretching of the entire uniaxial cell stretching region is simulated by COMSOL finite element simulation. Since the four borders of the rectangle are connected to the same chamber, the four borders of the rectangle region will be subjected to the same pulling force when the syringe is withdrawn. The surface stress distribution of the whole cell culture area under this pressure was obtained via simulation. The deeper the color is, the greater the stress. Therefore, combined with the displacement experiments and simulation results of the PS balls, the deformation of the film is approximately uniaxial deformation. This study demonstrated that even with common syringes and injectors acting as an external input, pure uniaxial direction stimulation was produced with our chip platform.

It is challenging to observe a change of only 10 μm on a horizontal plane with the naked eye or even with a low-magnification microscope. The aforementioned cell-stretching area, which reaches 2.5% at maximum extraction, is only 400 μm wide. Therefore, it is even more challenging to precisely and quantitatively measure the amount of stretching of the film at various extraction levels. Since the deformation of the film in the upper layer of the air cavity, which is connected to the sensitive area, leads to the stretching deformation of the film in the sensitive area when it is subjected to pressure, the measurement of the deformation of the film in the sensitive area can be converted into a measurement of the degree of tension and compression of the suspended film in the upper layer of the vacuum cavity^35,36^. The deflections of the suspended PDMS films embedded with fluorescent particles were observed and calibrated using a lateral microscope by Dou^21^ from the University of Toronto and Sato^37^ from Tokyo. Another method^34^ using electrical signals is crack-sensing to increase the sensitivity of the sensor, but this method has high complexity and low repeatability in manufacturing. Since lateral microscopy is very demanding in terms of equipment and environment, we used a microfluidic channel incorporated into the suspension film and liquid metal to obtain a highly accurate miniature in situ strain transducer^35,36^. This method allows us to obtain the magnitude of tensile deformation in the sensitive region utilizing an on-chip integrated sensor coupled to an external circuit.

**Fig. 2:**
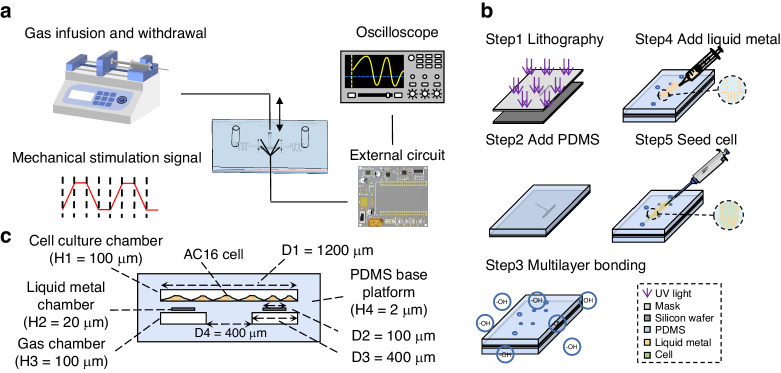
Experimental system and experimental flow chart. **a** A schematic of the components of the stain-sensor-on-chip. The injection valve is connected through the gas pipe and controls the uniform extraction and perfusion of gas in the vacuum chamber. The chip-integrated sensor connects to the external circuit to obtain signals and displays the deformation size of the sensitive area inside the chip through the oscilloscope. The chip can be placed above the objective lens of the microscope for observations. **b** Chip manufacturing process: Step 1, the use of soft lithography technology to manufacture the SU-8 mold; Step 2, curing according to the proportion of PDMS in the mold; Step 3, plasma treatment; Step 4, Galinstan injection into the liquid channel; Step 5, cells and culture medium injection, into the cell culture. **c** Cross-sectional diagram of the chip: the cell culture chamber layer, liquid metal layer, and vacuum chamber layer from top to bottom

The described in situ sensor on a chip is designed and fabricated based on an embedded Galinstan microfluidic channel with a height of 20 μm and a width of 50 μm (Fig. 2c and Fig. S2). The fabrication process is shown in detail in Fig. 2b and Fig. S1. The simulation diagram in Fig. 1f shows the displacement of the film above and below the liquid metal flow channel after the pressure is applied, and the bottom diagram in Fig. 1f shows the results of the membrane deformation after the above pressure. It can be seen from the simulation results that the cross-section will not change greatly, but because the liquid metal channel layer is a cavity below, the film will sag down with the liquid metal, and the length of the liquid metal will change greatly, so the resistance length will increase. According to the resistance calculation formula:

1 $$R=\frac{\rho L}{S}$$

where *R* is the resistance value of the sensor, *L* is the length of the flow path, *S* is the cross-section of the flow path, and *ρ* is the resistivity of the liquid metal is constant. As *L* increases l, *S* basically remains the same, and the resistance of the overall sensor increases during stretching.

The middle of the channel receives the largest deformation force, corresponding to the most significant strain variable *ε*_max_, given by

2 $${\varepsilon }_{\max }=\frac{3P{L}_{0}^{2}(1-{v}^{2})}{8{h}^{2}E}$$

The assumed values are *P*, the pressure exerted on the film; *L*_*0*_, the channel width; *h*, the diaphragm thickness; *v*, is the Poisson’s ratio; and *E*, is Young’s modulus. The sides around the rotor are fixed.

3 $${\varepsilon }_{\min }=0$$

Assuming that the strain gauge factor of the liquid metal equals 2, for a given input voltage *V*_in_, the theoretical output voltage *V*_out_ of the sensor is approximated as

4 $${V}_{\rm{out}}=0.75\frac{P{L}_{0}^{2}(1-{v}^{2})}{{h}^{2}E}{V}_{\rm{in}}$$

After calculation, it can be concluded that the pressure *P* and the voltage are directly proportional to each other, which is also supported by the simulation results in Fig. 2a. The above considerations are ideal states based on a number of assumptions of the calculation. The actual experimental results and theoretical calculations have some deviation but basically tend to be proportional to the relationship.

### On-chip strain sensing quantified stimulus amplitude

We established and assessed several critical performance characteristics in order to fully characterize the performance of the microfluidic diaphragm sensor. Sensitivity, linearity, reproducibility, and stability are some of these indications. In contrast to the linearity obtained from the sensitivity regression line, the sensitivity measures the effect of each unit of pressure on the sensor voltage. Since the use of sensors with resistive sensing mechanisms and temperature sensitivity is a drawback of liquid metals^38^, stability generally relates to retaining the same resistance at different temperatures^39^.

**Fig. 3:**
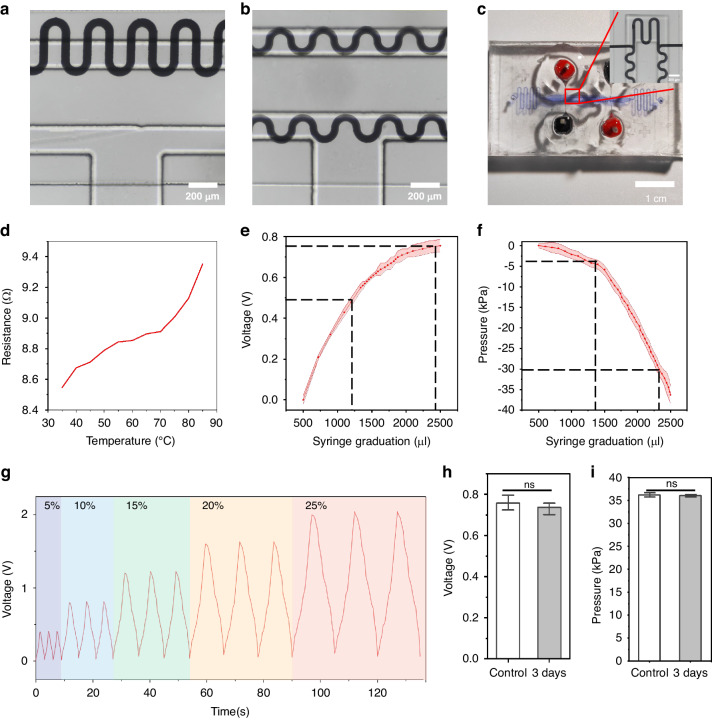
Sensor characterization and calibration. **a** Actual images of the single-arm sensor at 10× (scale bar: 200 μm). **b** Wheatstone bridge sensor at 10× (scale bar: 200 μm). **c** Real image of the sensor-on-chip (scale bar: 1 cm) and local magnification image under a 10× lens (scale bar: 200 μm.). **d** Relationships between the resistance value of the sensor and the temperature (*n* = 3). **e** Relationships between the output voltage of the sensor and the extraction flow rate of the syringe. **f** Relationships between the pressure inside the real air chamber and the extraction flow rate of the syringe. **g** Voltage changes in the output of the sensor at each strain amplitude (three cycles each). **h**, **i** Fatigue testing was performed by applying cyclic pressure for 3 days. Neither the sensor output voltage nor the pressure has a significantly changed

Figure 3a, b depicts two types of sensors: a single-arm sensor and a Wheatstone bridge sensor with built-in temperature correction. The single-arm sensor underwent temperature sensitivity testing, as depicted in Fig. 3d. Since liquid metals are sensitive to changes in ambient temperature, the lack of additional temperature correction has a substantial effect on the repeatability and stability of the sensor. By measuring the difference in resistance change, a bridge sensor with temperature compensation is created to eliminate the impact of the environment and temperature. The physical and localized perspective of the sensor is depicted in Fig. 3c. After that, we calibrated the connection between the external syringe extraction (perfusion) flow rate, the vacuum chamber pressure, and the relative change in sensor voltage (compared to the voltage output in the state of no external pressure being applied) (Fig. 3e, f). We determined the internal pressure of the vacuum chamber (5.55, 10.05, 16.47, 21.35, and 24.81 kPa) and the flow rate of the syringe withdrawal (200, 400, 600, 800, and 1000 μL). Figure 3g shows the real-time provenance voltage response for three cycles of extraction and perfusion at different tensile stresses (5%, 10%, 15%, 20%, and 25%), demonstrating the repeatability and stability of the sensor. In addition, fatigue testing was performed to assess the possible structural degradation of the sensor during prolonged use (Fig. 3h, i). Dynamic stimulation of the sensor for three days using maximum perfusion (25%) did not significantly change either the sensor voltage or the air pressure, which verified the stability of the device under long-term mechanical stimulation.

### Area and increment expression enhancement under mechanical stimulation

Since the heart beats continuously and independently, it is constantly exposed to various physiological stimuli, primarily mechanical stimuli^40,41^. As a result, mechanical stimuli generated by cardiomyocytes can be recognized as mechanical stimuli. We postulated that dynamic cardiomyocyte culture at various stimulation levels would classify cardiac cell disease and physiology^42,43^. Cardiomyocytes were cultivated in the abovementioned chip with integrated in situ sensors and subjected to calibrated 5%, 10%, 15%, 20 and 25% cyclic stretch conditions for 24 h to confirm the notion mentioned above that cardiomyocytes respond to externally applied mechanical stimuli. The findings revealed that the number of proliferating cells increased and that the size of the area changed slightly; however, more significantly, 15% mechanical stimulation promoted the overall alignment of the cells. This phenomenon was more obvious during long-term cultivation (60 h).

**Fig. 4:**
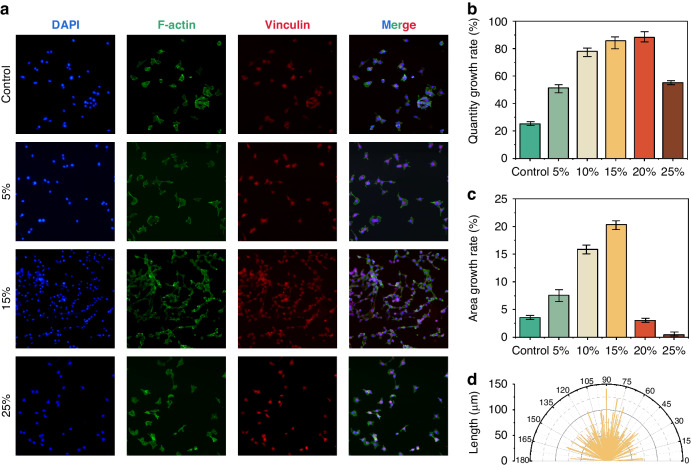
Mechanical stimulation affects the cell growth rate, area, and arrangement. **a** Immunofluorescence images (DAPI, actin, and vinculin) of cells at different stimulation loads (control, 5%, 15 and 25%). Scale bar: 100 μm. **b** Cell growth rate under different stimulation loads (control, 5%, 10%, 15%, 20 and 25%). **c** Cell area growth rate under different stimulation loads (control, 5%, 10%, 15%, 20 and 25%). **d** Cell orientation angle and cell longitudinal axis length under 15% mechanical stimulation

Using a microscope to observe the sensitive area inside the annular air cavity, we found that mechanical stimulation significantly promoted cell growth (Fig. S5 and Fig. 4). Stimulation of cardiomyocytes on the film with different strain amplitudes (no stimulation, 5%, 10%, 15%, 20%, and 25%) resulted in a rapid spreading of the cells and a corresponding increase in the cell area and number of cells. The changes in cell number and area after 24 h of continuous stimulation (control: cell number growth rate of 25.16%, cell area growth rate of 3.52%; 5%: cell number growth rate of 51.34%, cell area growth rate of 7.57%; 10%: cell number growth rate of 78.13%, cell area growth rate of 15.87%; 15%: cell number growth rate of 85.80% and cell area growth rate of 20.36%; 20%: cell number growth rate of 88.28% and cell area growth rate of 3.02%; 25%: cell number growth rate of 55.17% and cell area growth rate of 0.46%) are shown in Fig. S5. At 15% mechanical strain, there was a stable and rapid increase in cell number and area as the amplitude of mechanical stimulation increased. However, at higher mechanical strain levels, we noticed that the cells partially detached from the device mold and even started to cluster by death.

**Fig. 5:**
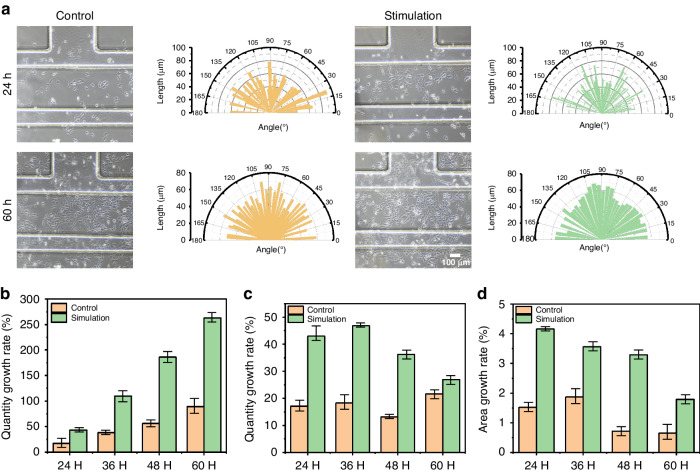
Growth rate, area, and arrangement of cells in long-term culture (60 h) under 15% mechanical stimulation. **a** Bright-field images (Scale bar: 100 μm) of the control group and stimulation group at different culture times (24 and 60 h). Statics chart of cell orientation angle and length of the cell’s vertical axis. **b** Cell number growth rate between the control group and the stimulation group at different culture times (24 h, 36 h, 48 h, and 60 h). **c** Comparison of cell area growth rate between the control group and the stimulation group at different culture times (24 h, 36 h, 48 h, and 60 h). **d** Cell area growth rate between the control group and the stimulation group at different culture times (24 h, 36 h, 48 h, and 60 h)

We evaluated the effect of mechanical stimulation on cell alignment by determining the angle and length of the long axis of the cells (Fig. S6 and Fig. 5). Compared with the control group, a certain degree of mechanical stimulation was able to align the length and angle of the cells (*n* = 99 cells in each case), and it has been shown that explicit mechanical strain leads to the reorganization of the various cell types along the direction of the strain. Some scholars have shown that mechanical strain causes various cells to realign along the direction of the strain, and for cardiomyocytes, the alignment behavior is regulated by mechanotransduction resulting in “strain avoidance^44,45^”. Through the above analysis, we can see a significant difference between the strains applied to our chip in the transverse and longitudinal directions, which is likely the reason for the rearrangement of the cardiomyocytes. After the cells were incubated for an extended period until the cell culture chamber was filled with cells under 15% stimulation, the consistency of our platform’s cell orientations became more obvious after 60 h of incubation.

The design of the vacuum cavity needs to be improved to add uniaxial control in various directions, devices that can apply dynamically controllable anisotropic biaxial strains to cells, and even devices with circumferentially oriented stresses to further investigate the impact of stress direction on cell alignment^46^. Fluorescence immunostaining is performed to further characterize the response of cardiomyocytes to stimuli. The design of multimodal built-in sensors is required to obtain more multidimensional information about the environment inside the chip (cell culture chamber) prior to further research on immediate testing^47^ of combined stimuli, such as electrical stimulation, to improve the simulation of the cellular environment.

## Conclusion

The research in this paper focused on the precise quantification of mechanical stimuli via embedded sensors in organ-on-a-chip. We developed a platform with an integrated vacuum chamber to provide precise uniaxial tensile control of the biochemical and mechanical environment experienced by the in vivo microenvironment. Sensitive liquid metal flexible sensors were integrated into the vacuum chamber to measure the deformation of the membrane in the sensitive region. The flexible strain sensors used a Wheatstone bridge design to increase sensitivity, while temperature self-compensation kept the sensors free of external bias. Mechanical stimulation was applied to cells inside the organ-on-a-chip with strain amplitudes ranging from 5 to 25% by using the abovementioned flexible sensors calibrated to the relationship between external stimulation pump flow, vacuum chamber pressure, and sensor voltage changes. By applying different amplitudes of stimulation to cardiomyocytes, our experimental results showed that the cell number and area gradually increased with increasing mechanical stimulation and gradually unified in terms of the consistency of cell alignment, which stabilized at 15%, with a more significant effect after 3 days of culturing. Therefore, our in situ strain sensor on a chip is a good platform for measuring the mechanical stress on cells. The work in this paper has made innovations in the internal environment sensing of organ-on-a-chips, which is expected to improve the precise control of the internal environment of microfluidic chips and organ-on-a-chip and promote the quantitative research on cell mechanical conduction results.

## Materials and methods

### Chip structure and working mechanisms

**Fig. 6:**
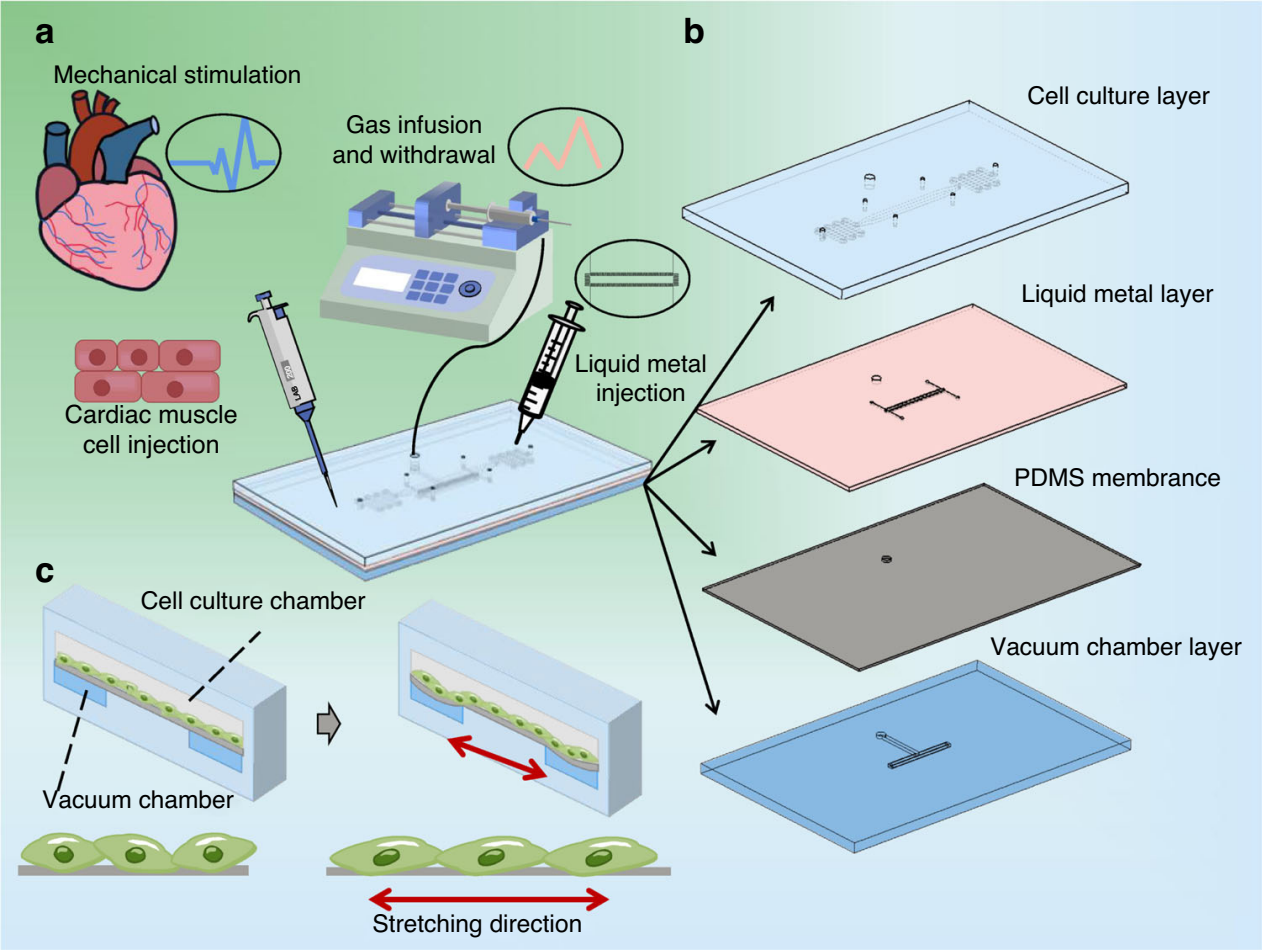
A schematic of the strain sensor on a chip. **a** Using organ-on-a-chip for cultured cardiomyocytes. The chip integrates a vacuum chamber to apply tensile stress to the cells to mimic the microenvironment in vivo; integrated sensor feedback quantifies cell deformation. **b** A cell culture layer, a liquid metal layer, a thin film layer, and a vacuum chamber layer can be assembled into an integrated chip that contains three microfluidic channels: a vacuum chamber, a liquid metal injection channel, and a cell culturing chamber. **c** Schematic diagram of a prestretch (left) and poststretch (right) stretch device. The diagram shows the cross-section and the working mechanism of the chip. When vacuum pressure is applied to the annular vacuum chamber, the wall of the annular vacuum chamber deflects outward, stretching the internal suspended film and the adherent cells on it. The cells on the membrane are stretched and relaxed repeatedly

We created a gas-chamber-surrounded chip with an integrated in situ sensor that consists of four layers: a cell culture layer, a liquid metal layer, a PDMS membrane layer, and a vacuum chamber layer (Fig. 6b). All of the aforementioned layers are made of PDMS (Sylgard-184, Dow), which has good light transmission characteristics and enables the microscopic examination of the chip’s interior structure and cells. The four layers of the chip are strongly bound together by oxygen plasma (Diener Electronic, Zepto). The cell culture layer features a long channel (length: 1 cm, width: 400 μm) flanked by through-holes (depth: 2 mm, radius: 1 mm) for injecting cell suspensions and cell culture medium. The bottom layer of the device contains the vacuum chamber, which also serves as the bottom of the cell culture chamber, allowing cells to attach and grow against the membrane surface and stretch when the gas is extracted (Fig. 6c). The liquid metal channel (height: 20 μm, width: 50 μm), embedded in the elastic film in the middle layer, is suspended above the vacuum chamber. The deformation of the suspended film inside can be precisely measured in real-time by an external circuit because the channel wrapped with liquid is sensitive to film deformation. An annular vacuum chamber (length: 1 cm, width: 600 μm) surrounds a central raised platform (length: 800 μm, width: 400 μm), which is the cell stretching area for observation. A cylindrical chamber (depth: 3 mm, radius: 1 mm) that is joined to a syringe (2 ml) and a micro control pump (LSP04-1A) is connected to the vacuum chamber. To mechanically stimulate adherent cells, the syringe was infused, and the vacuum chamber was withdrawn to deform the suspended film layer. The integrated sensor above the air cavity is connected to an external circuit to further process the signal, and finally, the amplitude of deformation in the sensitive area is displayed on the oscilloscope (Fig. 2a).

### Chip manufacturing

Figure 2b shows how the device was made. Soft lithography and negative adhesive (SU8 2050) were used to create PDMS molds for each layer of the channels. PDMS was then prepared in a 10:1 elastomer and hardener ratio and carefully mixed before being poured onto the molds. The molds were placed in a vacuum to remove air bubbles, which enhanced the device’s observation ability. Two grams of prepared liquid PDMS were placed in a dish and spun at 1000 rpm/min for 30 s using a spin coater (LEBO Science) to create a thin film of PDMS. The film was then dried at 65 °C for 2 h. After demolding, the surface was cleaned of dust, through holes were punched with a 1 mm hole punch, and each layer was then adhered. The liquid metal was injected into the liquid metal channel and sealed with a pin.

### Sensor manufacturing

In the suspended film above the gas chamber, embedded microfluidic channels were built up, and Galinstan (a eutectic alloy of gallium, indium, and tin) was injected into the channels. A flexible microfluidic sensor was created by filling the whole microfluidic channel with liquid metal because of its outstanding fluidity and conductivity. This flexible microfluidic sensor can then be connected to an external circuit to determine the degree of deformation of the film. After the preparation of the chip and the built-in sensor, the PDMS film was modified for cell culture.

### Piezoresistive Wheatstone bridge sensor principle

Volume changes caused by external mechanical deformation are the decisive factor in changes in conductor resistance. A rectangular sensitive region has four edges that are covered by four sets of serpentine sensor grids, which make up the embedded strain resistor. The four sensor grids (Fig. 1e) are connected end to end, and an equivalent Wheatstone bridge is created by using two nonadjacent segments for the reference voltage input and the other two pairs for the voltage output.

### Sensor quantification

A barometer was utilized to calculate the pressures corresponding to various syringe extractions into the vacuum cavity to calibrate the device. An additional part of the picture shows the tensile deformation of the film in the sensitive area for various extraction volumes (Fig. 1b). In COMSOL Multiphysics (Version 5.6, COMSOL AB, Stockholm, Sweden), finite element analysis was used to simulate the membrane’s displacement under strain in both the transverse and longitudinal directions (Fig. 1a). The inputs included the device’s dimensions, the suspended film’s characteristics, and the applied air pressure. It was expected that the PDMS material would have a Poisson’s ratio of 0.49 and be isoelastic in all directions.

The embedded liquid metal sensor collects resistance and voltage signals through a digital bridge (KEYSIGHT, E4980AL) and an external circuit coupled to an oscilloscope (Tektronix, TBS 1202B). This showed the correlation between the sensor voltage and gas extraction volume. The relationship between the sensor voltage and the tensile force applied to the film in the sensitive area was finally calibrated by combining the pressure–volume curve and the voltage–volume curve.

### Cell culture

Cells were grown in low-glucose DMEM (Corning) supplemented with 10% fetal bovine serum (FBS, Gemini Bio Products), at 37 °C with 5% CO_2_ for experiments employing human cardiomyocyte (AC16), an immortalized organelle. The cells (suspension at 0.8 × 10^6^ cells/ml in a volume of 10 μL) were added to the cell culture chambers after Laminin (Gibco) was added to the chambers to encourage cell attachment and spreading. This resulted in low-density monolayer cells that were ready for mechanical stimulation after 10 h.

### Cell imaging and immunofluorescence staining

Immunofluorescence staining of AC16 cardiomyocytes was performed to determine how mechanical stimulation affected cell maturation (Fig. S3). The cells were fixed with 4% paraformaldehyde (PFA) and permeabilized with 0.1% (w/v) Triton X-100 for 10 min after stimulation for 24 h (60 h for long-term culture). To prevent nonspecific antibody binding after permeabilization, the cells were blocked with antibody diluent (5% goat serum in TBST) for 90 min. The cells were incubated overnight at 4 °C with primary antibodies. Nuclei and intermediate filaments were stained with an anti-vinculin antibody [EPR3776] and the cytoskeletal marker phalloidin (Actin), respectively. Subsequently, the cells were incubated with secondary antibodies for 2 h at room temperature. Alexa Fluor 594-conjugated secondary antibodies (goat anti-rabbit IgG, ab150077, Abcam) were used for the visualization of microtubules and intermediate filaments, respectively. The above antibodies were diluted 1:1000 in antibody dilution buffer. To stain the microfilaments, the cells were incubated with phalloidin-tetramethylrhodamine isothiocyanate (TRITC, Sigma, diluted 3.5:500 in PBS) solution for 30 min at room temperature. Additionally, 4′,6-diamino-2-phenylindole (DAPI, 41002, Beyotime, diluted 1:1000 in PBS) was used to stain the cell nuclei. Nikon microscope (Nikon) fluorescence photos were taken, and ImageJ was used for analysis. The Morpholibj plugin for ImageJ was used for image processing throughout the analysis of the images. Cellulose was used to aid in the graphical segmentation of cell bright field images to obtain more accurate findings.

### Statistical analysis

All studies in this study using various batches of devices or cells were carried out in at least triplicate, and the results are shown as the mean ± standard error. Differences among the three groups were examined by one-way ANOVA. A *p*-value < 0.05 indicated statistical significance (ns = not significant).

### Supplementary information

Supplemental Material
