# Supplementary material for: Strain sensor on a chip for quantifying the magnitudes of tensile stress on cells
Source: Microsyst Nanoeng. 2024 Jun 25;10:88. doi: 10.1038/s41378-024-00719-z (PMC11196625; doi:10.1038/s41378-024-00719-z)
Supplement: Supplementary file 1 — Supplemental Material [file 41378_2024_719_MOESM1_ESM.doc]

# Strain Sensor on a Chip for Quantifying the Magnitudes of Tensile Stress on Cells

**Authors:** Yuyin Zhang^1^, Yue Wang^2,5*^, Hongze Yin^1^, Jiahao Wang^1^, Na Liu^1,3,4^, Songyi Zhong^1,2^, Long Li^1,3^, Quan Zhang^1,2,3^ and Tao Yue^1,2,3,4*^

**Affiliations:**

^1^School of Mechatronics Engineering and Automation, Shanghai University, Shanghai, China

^2^School of Future Technology, Shanghai University, Shanghai, China

^3^Shanghai Key Laboratory of Intelligent Manufacturing and Robotics, Shanghai University, Shanghai, China

^4^Shanghai Institute of Intelligent Science and Technology, Tongji University, Shanghai, China

^5^Key Laboratory of Advanced Manufacturing Technology of Zhejiang Province

* Correspondence to: Tao Yue (tao_yue@shu.edu.cn)

Yue Wang (yue_wang@shu.edu.cn)

Address:

99 Shangda Road, BaoShan District

Shanghai University


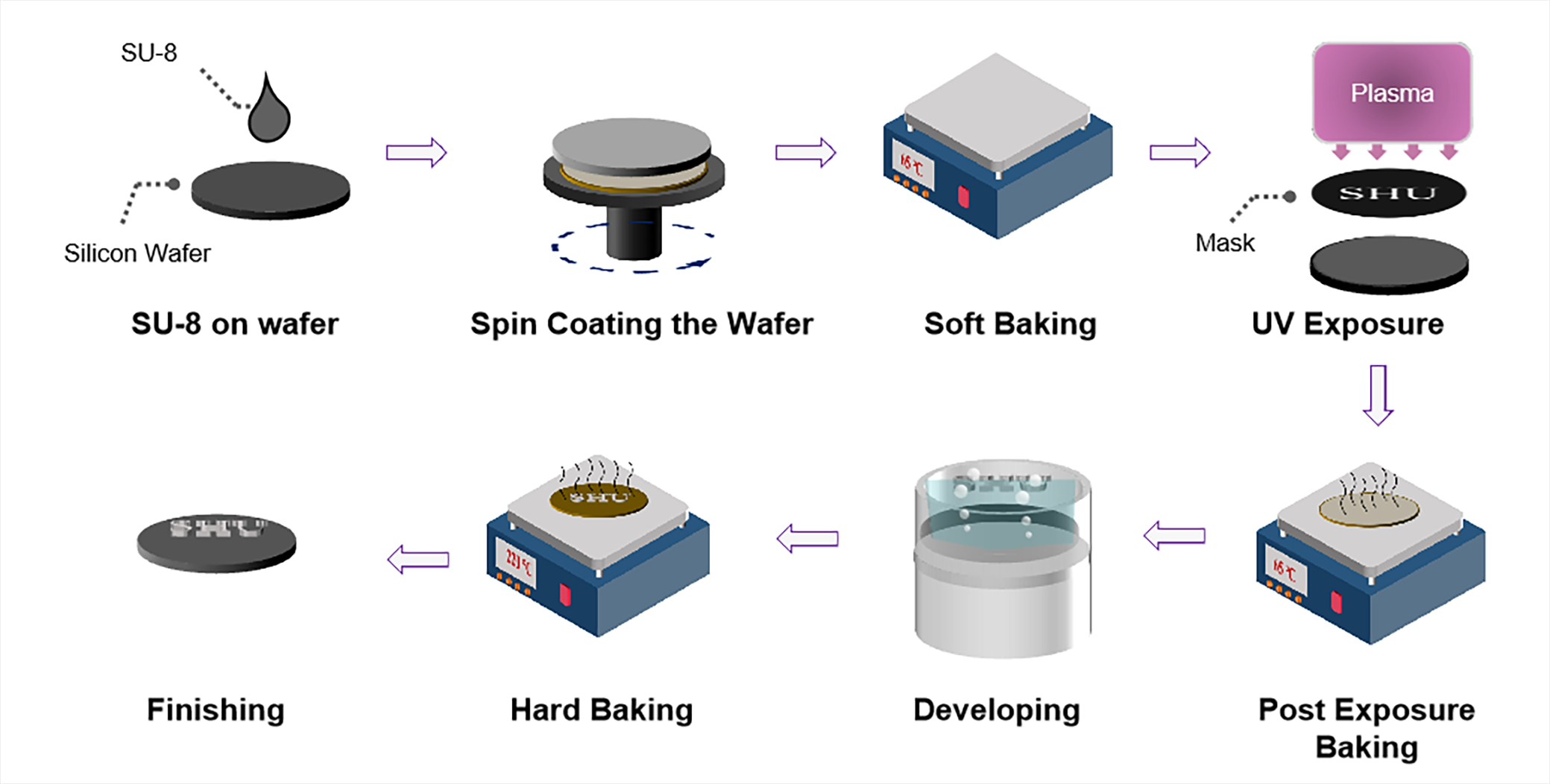


**Figure. S1** Fabrication processes of making a chip by using soft lithography.

Use soft lithography technology to make chips. Seven steps of soft lithography to make chip mask are shown in Figure S1.

Step 1: Add an appropriate amount of SU-8 to a clean silicon wafer.

Step 2: SU-8 on the silicon wafer is than spread by rotating the silicon wafer. The speed depends on the thickness of the channel to be lithographed.

Step 3: Soft breaking at 95 °C semi-solidfied the SU-8.

Step 4: Cover the designed mask on the SU-8 surface and exposure under the ultraviolet light. The transparent area of the mask will be cured.

Step 5: Post exposure baking at 95 °C for more robust lithography.

Step 6: Use the developer to clean the excess SU-8 on the silicon chip.

Step 7: Hard breaking at 220°C to make the lithography stronger.


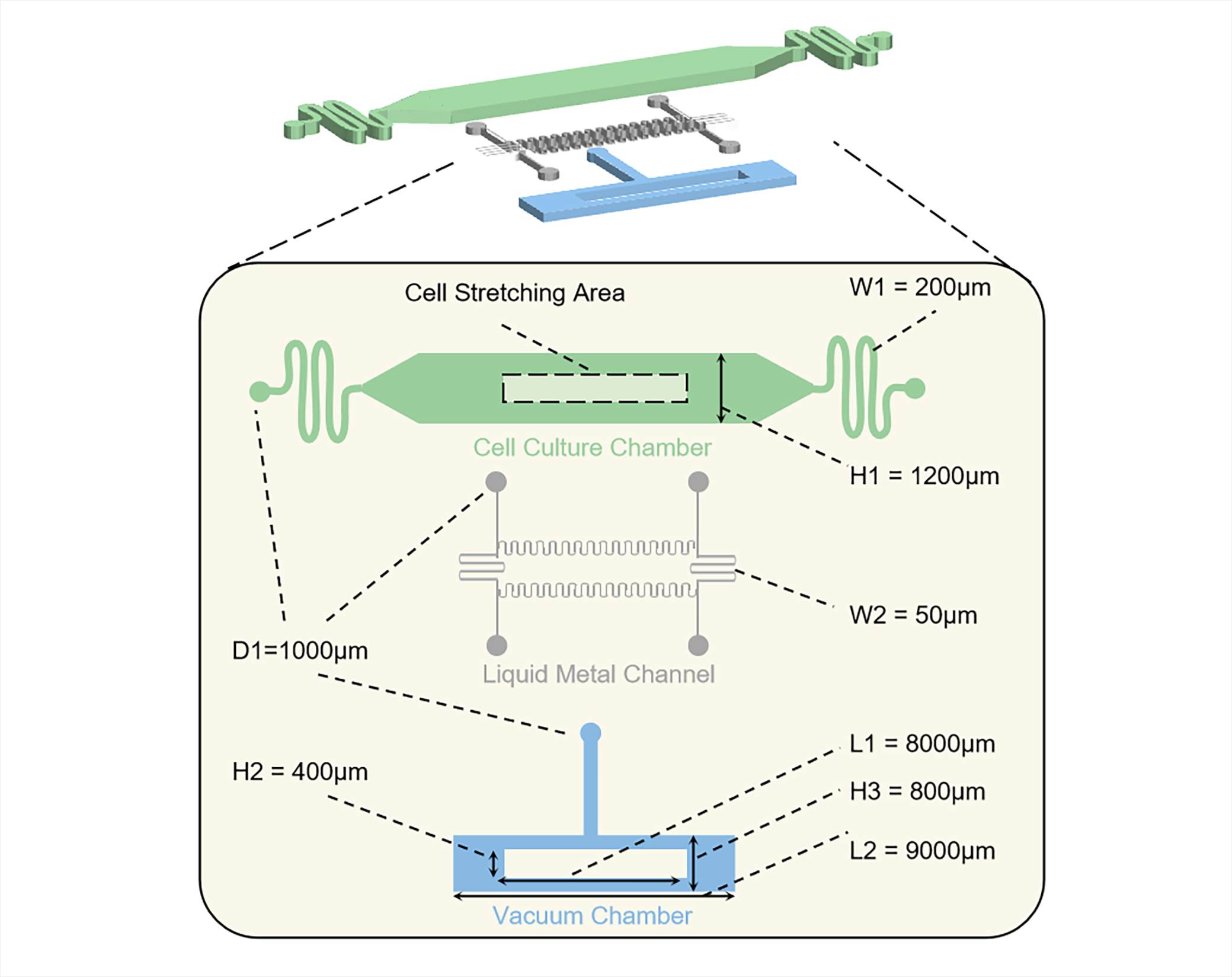


**Figure. S2** Layout of each layers. Specific data for cell culture layer, liquid metal layer and vacuum chamber layer.

Figure S2 shows the layout of each layer. The uppermost cell culture layer includes a long culture chamber and a serpentine tube (200 μm in width) for slowly injecting cell suspension. The middle layer is a serpentine channel with a width of 50 μm. The channels are connected at the end. There are four interfaces at the four ends for connecting wires.


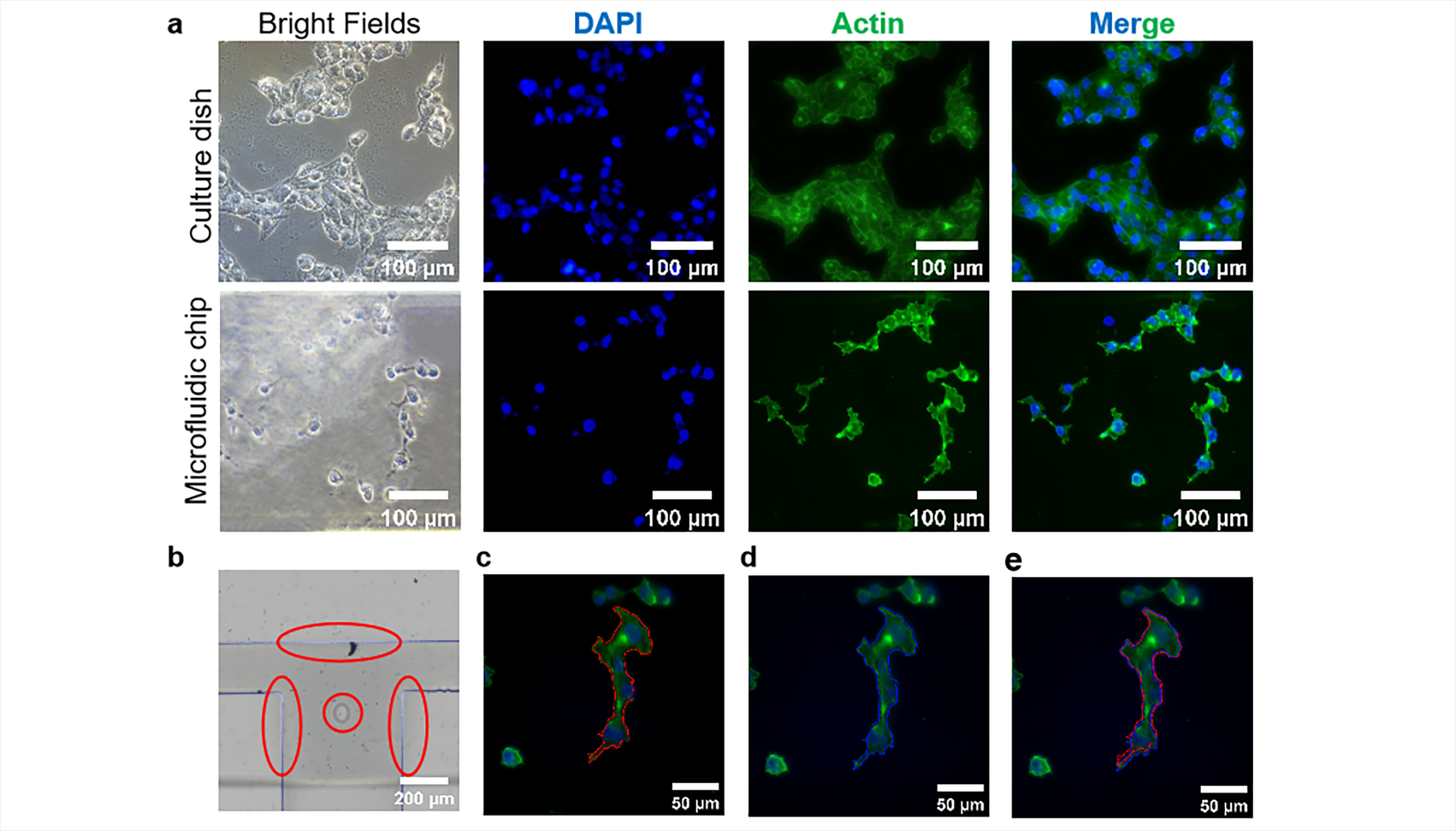


**Figure. S3** (a) Images of cardiomyocytes cultured in a dish under a 10x objective, including bright field images, DAPI nuclear staining, actin skeleton staining and merged images. Images of cardiomyocytes cultured in a chip under a 10x objective, bright field images, DAPI nuclear staining, actin skeleton staining and merged images. (b) Deformation of the vacuum chamber under a 10x objective. After gas extraction, the wall of the gas chamber will be slightly inclined, and the upper and lower layers of the gas chamber was touched. (c) The red outline is a set of static cells. (d) The blue outline is a set of stretched cells. (e) The blue-red combined outline shows how the cells changed before and after stretching.


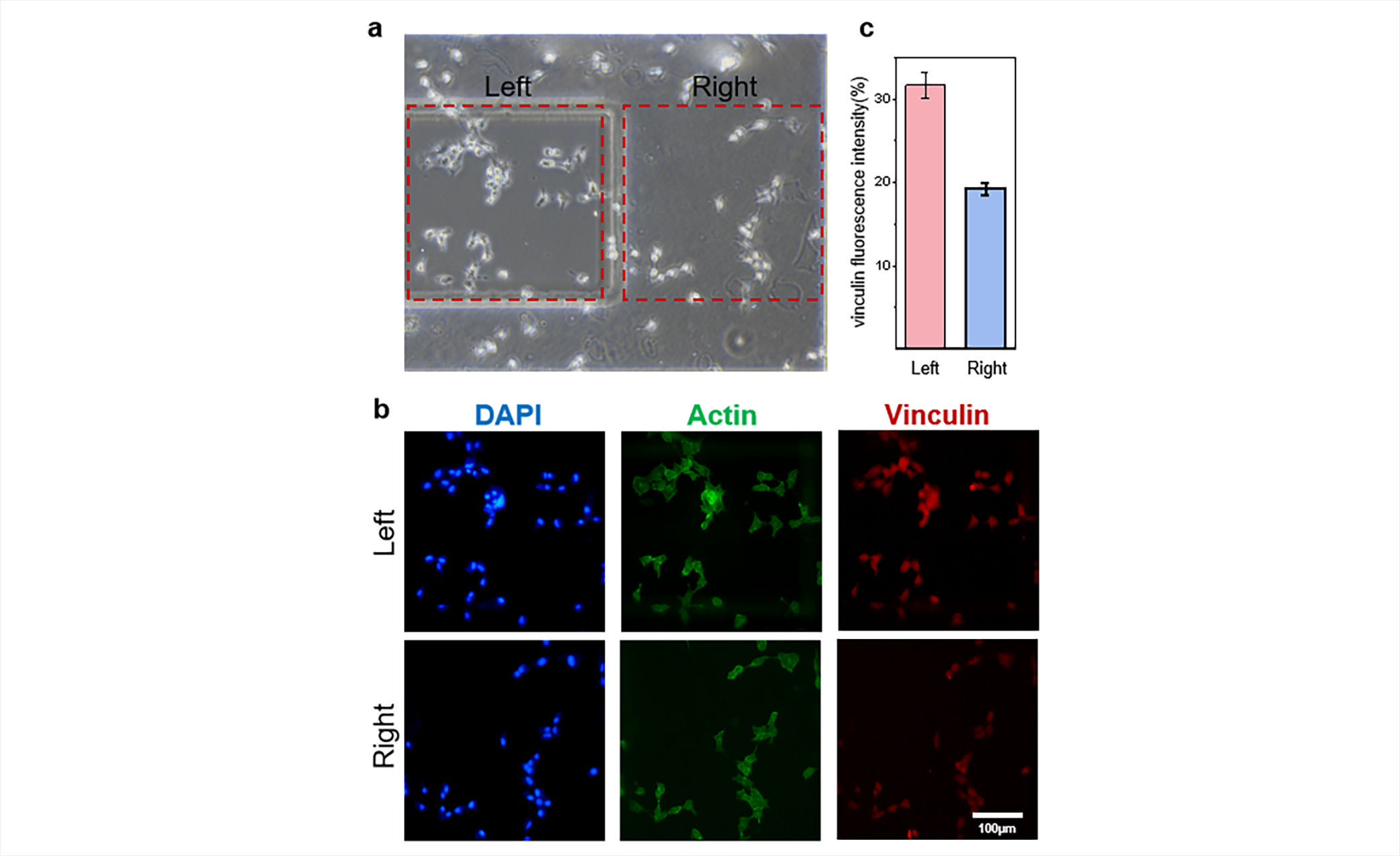


**Figure. S4** Comparison of stretched areas and other cell culture areas. (a) A bright field image containing the stretched area and other areas. The area in the red box on the left is the area being stretched; the area in the red box on the right is the static area. (b) For cells in the left and right areas, immunofluorescence staining (DAPI, actin and vinculin) was performed one day after stretching (Scale bar: 100 μm). (c) Statistics of immunofluorescence intensity of vinculin in the left and right areas (three sets of data are used for statistics, each set of data is calculated in a square area with an area of 400 μm).


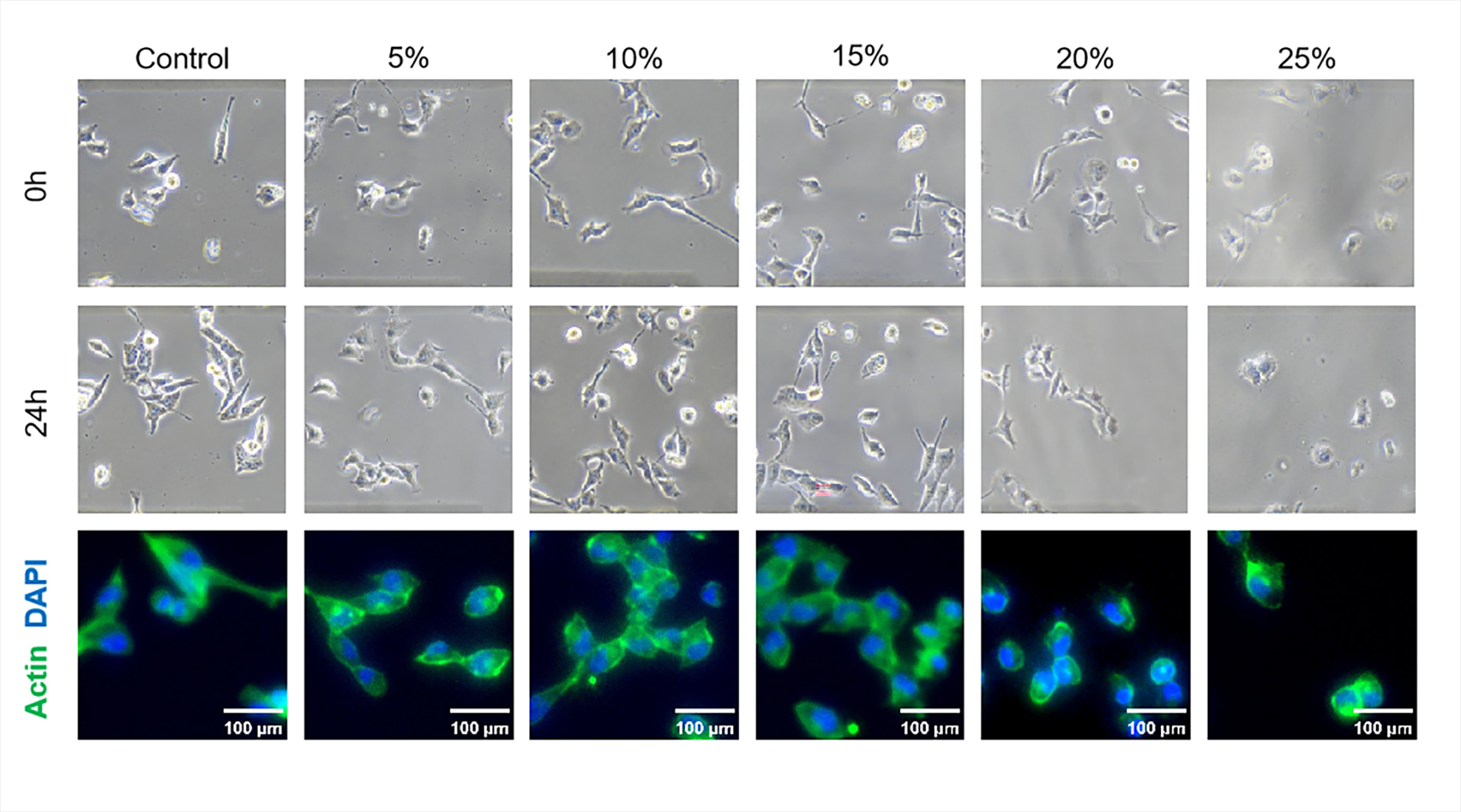


**Figure. S5** Bright-field images of cells at different stimulation loads (control, 5%, 10%, 15%, 20% and 25%), before and after stimulation for 24 hours. Immunofluorescence images (Actin and DAPI) of cells at different stimulation loads (control, 5%, 10%, 15%, 20% and 25%). Scale bar: 100 μm.


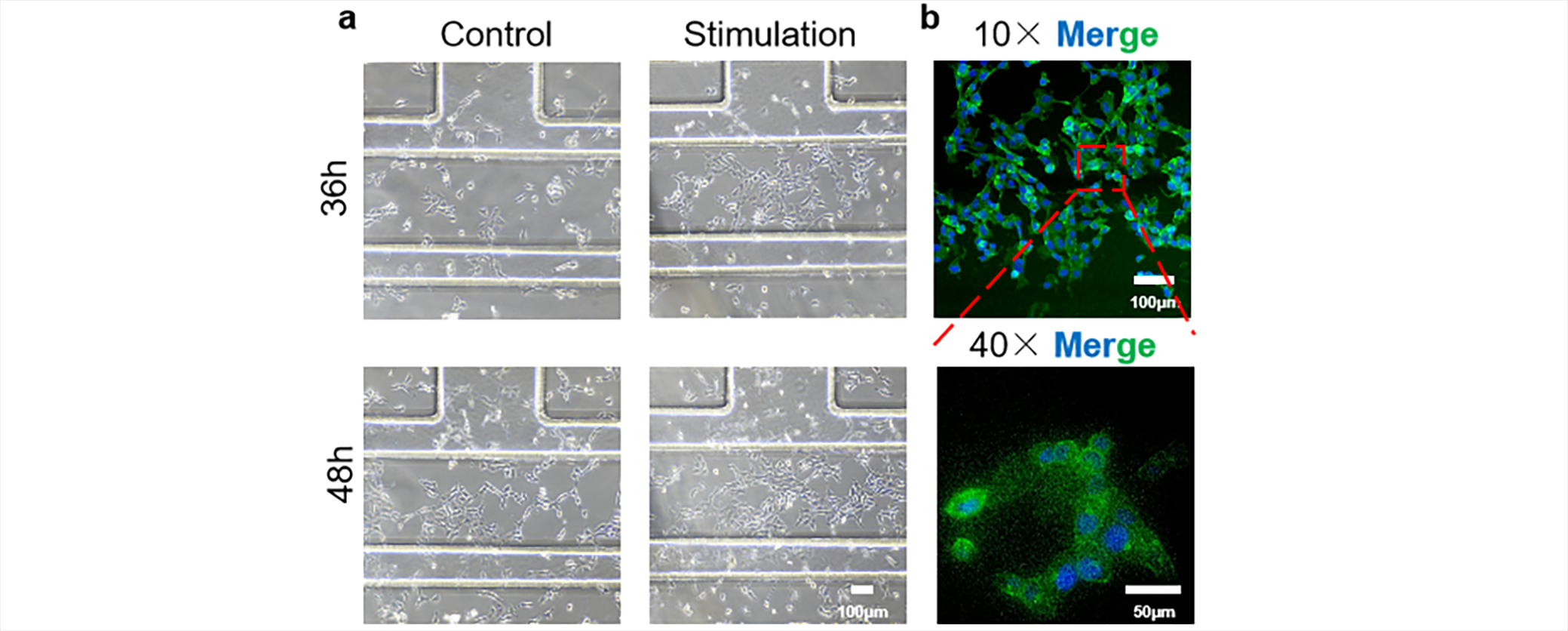
**Figure. S6** (a) Bright field images of the control group and stimulation group at different culture times (36 and 48 hours). (b) Immunofluorescence staining images (Actin and DAPI) at 60 hours of 15% stimulations at 10x (Scale bar: 100 μm) and 40x local magnification images (Scale bar: 50μm).
